# Piperazine-1,4-diium 2-(carb­oxy­meth­yl)-2-hy­droxy­butane­dioate monohydrate

**DOI:** 10.1107/S1600536810030151

**Published:** 2010-07-31

**Authors:** Ling-Li Liu

**Affiliations:** a Renmin Hospital of Wuhan University, Wuhan 430060, People’s Republic of China

## Abstract

In the crystal structure of the title compound, C_4_H_12_N_2_^2+^·C_6_H_6_O_7_^2−^·H_2_O, the cations, anions and water mol­ecules are linked by inter­molecular N—H⋯O, O—H⋯O and weak C—H⋯O hydrogen bonds into a three-dimensional network. An intra­molecular O—H⋯O inter­action occurs in the dianion.

## Related literature

For background to the applications of organic salts as pharmaceuticals, see: Du *et al.* (2009 ▶); Skovsgaard & Bond (2009 ▶); Yathirajan *et al.* (2005 ▶).
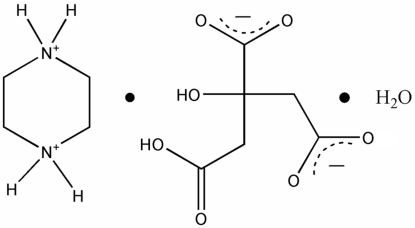


## Experimental

### 

#### Crystal data

C_4_H_12_N_2_^2+^·C_6_H_6_O_7_^2−^·H_2_O
*M* *_r_* = 296.28
Monoclinic, 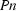

*a* = 9.2055 (12) Å
*b* = 6.8314 (9) Å
*c* = 11.2443 (14) Å
β = 112.047 (2)°
*V* = 655.41 (15) Å^3^
*Z* = 2
Mo *K*α radiation
μ = 0.13 mm^−1^
*T* = 298 K
0.20 × 0.10 × 0.10 mm

#### Data collection

Bruker SMART APEX CCD area-detector diffractometer
Absorption correction: multi-scan (*SADABS*; Sheldrick, 1996 ▶) *T*_min_ = 0.964, *T*_max_ = 0.987
4284 measured reflections
1605 independent reflections
1597 reflections with *I* > 2σ(*I*)
*R*_int_ = 0.088

#### Refinement

*R*[*F* ^2^ > 2σ(*F* ^2^)] = 0.045
*wR*(*F* ^2^) = 0.124
*S* = 0.84
1605 reflections
205 parameters
15 restraints
H atoms treated by a mixture of independent and constrained refinement
Δρ_max_ = 0.34 e Å^−3^
Δρ_min_ = −0.49 e Å^−3^

### 

Data collection: *SMART* (Bruker, 2001 ▶); cell refinement: *SAINT* (Bruker, 2001 ▶); data reduction: *SAINT*; program(s) used to solve structure: *SHELXS97* (Sheldrick,2008 ▶); program(s) used to refine structure: *SHELXL97* (Sheldrick,2008 ▶); molecular graphics: *PLATON* (Spek, 2009 ▶); software used to prepare material for publication: *PLATON*.

## Supplementary Material

Crystal structure: contains datablocks global, I. DOI: 10.1107/S1600536810030151/lh5096sup1.cif

Structure factors: contains datablocks I. DOI: 10.1107/S1600536810030151/lh5096Isup2.hkl

Additional supplementary materials:  crystallographic information; 3D view; checkCIF report

## Figures and Tables

**Table 1:** Hydrogen-bond geometry (Å, °)

*D*—H⋯*A*	*D*—H	H⋯*A*	*D*⋯*A*	*D*—H⋯*A*
N1—H1*A*⋯O3^i^	0.85 (2)	2.48 (3)	3.068 (3)	126 (3)
N1—H1*A*⋯O4^i^	0.85 (2)	1.99 (3)	2.764 (3)	150 (3)
N1—H1*B*⋯O1	0.86 (2)	1.95 (2)	2.806 (3)	174 (4)
N2—H2*A*⋯O5^ii^	0.86 (2)	1.86 (2)	2.706 (2)	167 (4)
N2—H2*B*⋯O1^iii^	0.86 (2)	1.99 (2)	2.804 (3)	159 (4)
O3—H3*C*⋯O2	0.87 (3)	1.92 (4)	2.685 (3)	147 (4)
O6—H6*C*⋯O5^iv^	0.87 (3)	1.82 (3)	2.671 (3)	167 (5)
O8—H8*C*⋯O2	0.87 (8)	2.00 (4)	2.798 (4)	151 (8)
O8—H8*D*⋯O1^iv^	0.87 (8)	2.15 (4)	3.003 (5)	165 (10)
C1—H1*C*⋯O7^ii^	0.97	2.47	3.391 (3)	159
C3—H3*A*⋯O7^v^	0.97	2.58	3.394 (3)	142
C3—H3*B*⋯O8^vi^	0.97	2.41	3.344 (7)	161
C4—H4*B*⋯O5^ii^	0.97	2.58	3.274 (3)	128
C6—H6*A*⋯O6^vi^	0.97	2.57	3.338 (3)	136

Symmetry codes: (i) 
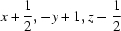
; (ii) 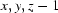; (iii) 
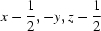
; (iv) 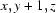; (v) 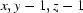; (vi) 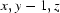.

**Fig. 1.**
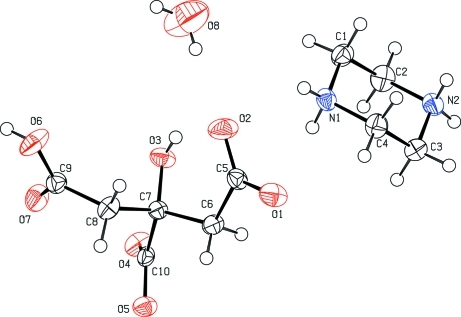
The asymmetric unit of (I), showing the atom-numbering scheme. Displacement ellipsoids are drawn at the 50% probability level and H atoms are shown as small spheres of arbitrary radii.

**Fig. 2.**
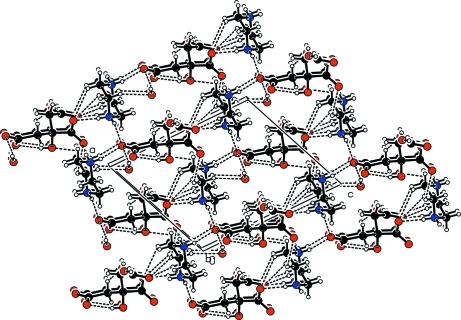
Part of crystal structure showing hydrogen bonds as dashed lines (I).

## supplementary crystallographic information

### Comment

Molecular adducts or cocrystals are widely applied in the fields of pharmaceuticals (Yathirajan, *et al.*, 2005, Skovsgaard & Bond, 2009, Du *et al.*, 2009). Herein, the crystal structure of the title compound (I) is reported.

The asymmetic unit of (I) composed of one piperazinium divalent cation, one citrate divalent anion and one solvent water molecule (see Fig.1). In the crystal structure, the piperazinium cations, citrate anions and water molecules are linked by intermolecular N—H···O, O—H..O and weak C—H···O hydrogen bonds (Table 1) into a three-dimensional network (Fig.2).

### Experimental

All the reagents and solvents were used as obtained without further purification. Equivalent molar amount of piperazine (0.2 mmol, 17.2 mg) and citric acid (0.2 mmol, 42.1 mg) were dissolved in 10 ml 95% methanol. The mixture was stirred for ten minutes at ambient temperature and then filtered. The resulting colorless solution was kept in air for three week. Block-shaped crystals of (I) suitable for single-crystal X-ray diffraction analysis were grown by slow evaporation of the solution at the bottom of the vessel.

### Refinement

In the absence of anomalous dispersion effects the Fridel pairs were merged. H atoms bonded to C atoms were positioned geometrically with C–H = 0.97Å and refined in a riding-model approximation with *U*_iso_(H) = 1.2*U*_eq_(C)]. H atoms bonded to N and O atoms were found from the difference maps and the N—H and O—H distances were refined using commands of 'SADI' and 'DFIX' in SHELXL (Sheldrick, 2008). The *U*_iso_(H) values were set to  1.2 and 1.5 times of 1.2*U*_eq_(N) and 1.5*U*_eq_(O), respectively.

### Crystal data

**Table:**

C_4_H_12_N_2_^2+^·C_6_H_6_O_7_^2^^−^·H_2_O	*F*(000) = 316
*M**_r_* = 296.28	*D*_x_ = 1.501 Mg m^−^^3^
Monoclinic, *P**n*	Mo *K*α radiation, λ = 0.71073 Å
Hall symbol: P -2yac	Cell parameters from 3551 reflections
*a* = 9.2055 (12) Å	θ = 2.4–28.2°
*b* = 6.8314 (9) Å	µ = 0.13 mm^−^^1^
*c* = 11.2443 (14) Å	*T* = 298 K
β = 112.047 (2)°	Block, colorless
*V* = 655.41 (15) Å^3^	0.20 × 0.10 × 0.10 mm
*Z* = 2	

### Data collection

**Table:**

Bruker SMART APEX CCD area-detector diffractometer	1605 independent reflections
Radiation source: fine focus sealed Siemens Mo tube	1597 reflections with *I* > 2σ(*I*)
graphite	*R*_int_ = 0.088
0.3° wide ω exposures scans	θ_max_ = 28.2°, θ_min_ = 2.5°
Absorption correction: multi-scan (*SADABS*; Sheldrick, 1996)	*h* = −12→12
*T*_min_ = 0.964, *T*_max_ = 0.987	*k* = −9→7
4284 measured reflections	*l* = −11→14

### Refinement

**Table:**

Refinement on *F*^2^	Primary atom site location: structure-invariant direct methods
Least-squares matrix: full	Secondary atom site location: difference Fourier map
*R*[*F*^2^ > 2σ(*F*^2^)] = 0.045	Hydrogen site location: inferred from neighbouring sites
*wR*(*F*^2^) = 0.124	H atoms treated by a mixture of independent and constrained refinement
*S* = 0.84	*w* = 1/[σ^2^(*F*_o_^2^) + (0.1299*P*)^2^ + 0.0532*P*] where *P* = (*F*_o_^2^ + 2*F*_c_^2^)/3
1605 reflections	(Δ/σ)_max_ < 0.001
205 parameters	Δρ_max_ = 0.34 e Å^−^^3^
15 restraints	Δρ_min_ = −0.49 e Å^−^^3^

### Special details

**Table:**

Geometry. All e.s.d.'s (except the e.s.d. in the dihedral angle between two l.s. planes) are estimated using the full covariance matrix. The cell e.s.d.'s are taken into account individually in the estimation of e.s.d.'s in distances, angles and torsion angles; correlations between e.s.d.'s in cell parameters are only used when they are defined by crystal symmetry. An approximate (isotropic) treatment of cell e.s.d.'s is used for estimating e.s.d.'s involving l.s. planes.
Refinement. Refinement of *F*^2^ against ALL reflections. The weighted *R*-factor *wR* and goodness of fit *S* are based on *F*^2^, conventional *R*-factors *R* are based on *F*, with *F* set to zero for negative *F*^2^. The threshold expression of *F*^2^ > σ(*F*^2^) is used only for calculating *R*-factors(gt) *etc*. and is not relevant to the choice of reflections for refinement. *R*-factors based on *F*^2^ are statistically about twice as large as those based on *F*, and *R*- factors based on ALL data will be even larger.

### Fractional atomic coordinates and isotropic or equivalent isotropic displacement parameters (Å^2^)

**Table:**

	*x*	*y*	*z*	*U*_iso_*/*U*_eq_	
C1	−0.0340 (3)	0.4003 (3)	−0.1528 (2)	0.0297 (4)	
H1C	−0.0235	0.4467	−0.2307	0.036*	
H1D	−0.0636	0.5106	−0.1124	0.036*	
C2	−0.1605 (3)	0.2459 (4)	−0.1861 (3)	0.0332 (5)	
H2C	−0.1796	0.2105	−0.1097	0.040*	
H2D	−0.2570	0.2982	−0.2484	0.040*	
C3	0.0376 (3)	−0.0130 (3)	−0.1483 (2)	0.0305 (5)	
H3A	0.0673	−0.1265	−0.1859	0.037*	
H3B	0.0251	−0.0540	−0.0701	0.037*	
C4	0.1648 (3)	0.1419 (4)	−0.1174 (2)	0.0303 (5)	
H4A	0.2618	0.0903	−0.0553	0.036*	
H4B	0.1826	0.1754	−0.1946	0.036*	
C5	0.0510 (2)	0.2958 (3)	0.2147 (2)	0.0246 (4)	
C6	0.0104 (3)	0.2231 (3)	0.3269 (2)	0.0265 (4)	
H6A	0.0993	0.1501	0.3846	0.032*	
H6B	−0.0768	0.1325	0.2937	0.032*	
C7	−0.0330 (2)	0.3809 (3)	0.40527 (19)	0.0199 (4)	
C8	0.1144 (3)	0.4982 (3)	0.4852 (2)	0.0262 (4)	
H8A	0.1838	0.4157	0.5529	0.031*	
H8B	0.1697	0.5375	0.4307	0.031*	
C9	0.0719 (3)	0.6782 (3)	0.5437 (2)	0.0252 (4)	
C10	−0.0977 (2)	0.2788 (3)	0.49726 (19)	0.0214 (4)	
N1	0.1189 (2)	0.3207 (3)	−0.06442 (18)	0.0258 (4)	
H1A	0.194 (3)	0.403 (4)	−0.044 (4)	0.031*	
H1B	0.121 (4)	0.286 (5)	0.010 (2)	0.031*	
N2	−0.1133 (2)	0.0681 (3)	−0.23994 (18)	0.0265 (4)	
H2A	−0.094 (4)	0.085 (6)	−0.308 (3)	0.032*	
H2B	−0.183 (4)	−0.019 (4)	−0.248 (4)	0.032*	
O1	0.1237 (2)	0.1775 (3)	0.17088 (18)	0.0372 (5)	
O2	0.0071 (3)	0.4609 (3)	0.1684 (2)	0.0461 (6)	
O3	−0.1506 (2)	0.5083 (3)	0.32461 (16)	0.0297 (4)	
H3C	−0.122 (5)	0.535 (7)	0.261 (4)	0.045*	
O4	−0.2272 (2)	0.3241 (2)	0.49788 (19)	0.0315 (4)	
O5	−0.0096 (2)	0.1479 (3)	0.56970 (16)	0.0303 (4)	
O6	0.1374 (3)	0.8387 (3)	0.5218 (2)	0.0423 (5)	
H6C	0.102 (6)	0.942 (6)	0.547 (5)	0.063*	
O7	−0.0127 (3)	0.6765 (3)	0.60374 (19)	0.0368 (4)	
O8	−0.0953 (5)	0.8366 (6)	0.0753 (6)	0.0923 (14)	
H8C	−0.032 (9)	0.739 (7)	0.107 (10)	0.139*	
H8D	−0.039 (10)	0.938 (7)	0.114 (9)	0.139*	

### Atomic displacement parameters (Å^2^)

**Table:**

	*U*^11^	*U*^22^	*U*^33^	*U*^12^	*U*^13^	*U*^23^
C1	0.0348 (10)	0.0246 (10)	0.0330 (11)	0.0013 (9)	0.0165 (8)	−0.0009 (8)
C2	0.0289 (10)	0.0379 (12)	0.0375 (12)	0.0018 (9)	0.0177 (9)	−0.0036 (10)
C3	0.0352 (11)	0.0250 (10)	0.0302 (10)	−0.0009 (8)	0.0112 (9)	0.0010 (8)
C4	0.0260 (9)	0.0318 (11)	0.0322 (11)	−0.0007 (8)	0.0097 (8)	−0.0022 (9)
C5	0.0272 (9)	0.0293 (9)	0.0195 (8)	0.0015 (8)	0.0112 (7)	0.0005 (7)
C6	0.0391 (11)	0.0221 (9)	0.0259 (10)	0.0037 (8)	0.0211 (9)	0.0018 (7)
C7	0.0229 (8)	0.0193 (8)	0.0203 (8)	0.0014 (7)	0.0115 (7)	0.0013 (6)
C8	0.0240 (8)	0.0227 (8)	0.0351 (10)	−0.0027 (7)	0.0147 (7)	−0.0024 (8)
C9	0.0276 (10)	0.0238 (9)	0.0252 (10)	−0.0019 (7)	0.0110 (8)	−0.0007 (7)
C10	0.0294 (9)	0.0171 (8)	0.0213 (8)	−0.0024 (7)	0.0138 (7)	−0.0031 (6)
N1	0.0304 (9)	0.0286 (9)	0.0215 (8)	−0.0066 (7)	0.0132 (7)	−0.0017 (6)
N2	0.0295 (8)	0.0292 (9)	0.0225 (8)	−0.0087 (7)	0.0116 (7)	−0.0001 (7)
O1	0.0466 (10)	0.0427 (10)	0.0324 (9)	0.0174 (8)	0.0264 (8)	0.0075 (7)
O2	0.0781 (16)	0.0326 (9)	0.0428 (11)	0.0132 (10)	0.0402 (11)	0.0136 (8)
O3	0.0319 (7)	0.0340 (8)	0.0255 (7)	0.0119 (6)	0.0134 (6)	0.0082 (6)
O4	0.0296 (8)	0.0290 (8)	0.0442 (10)	0.0006 (6)	0.0234 (7)	0.0021 (7)
O5	0.0422 (9)	0.0269 (7)	0.0294 (8)	0.0073 (6)	0.0220 (7)	0.0057 (6)
O6	0.0593 (12)	0.0196 (8)	0.0672 (14)	−0.0016 (7)	0.0456 (12)	−0.0006 (7)
O7	0.0514 (11)	0.0315 (9)	0.0396 (10)	−0.0068 (7)	0.0307 (9)	−0.0060 (6)
O8	0.076 (2)	0.0599 (19)	0.123 (4)	−0.0029 (17)	0.017 (2)	0.014 (2)

### Geometric parameters (Å, °)

**Table:**

C1—N1	1.488 (3)	C6—H6B	0.9700
C1—C2	1.510 (3)	C7—O3	1.420 (2)
C1—H1C	0.9700	C7—C10	1.540 (3)
C1—H1D	0.9700	C7—C8	1.541 (3)
C2—N2	1.492 (3)	C8—C9	1.514 (3)
C2—H2C	0.9700	C8—H8A	0.9700
C2—H2D	0.9700	C8—H8B	0.9700
C3—N2	1.491 (3)	C9—O7	1.207 (3)
C3—C4	1.519 (3)	C9—O6	1.318 (3)
C3—H3A	0.9700	C10—O4	1.235 (3)
C3—H3B	0.9700	C10—O5	1.274 (3)
C4—N1	1.488 (3)	N1—H1A	0.85 (2)
C4—H4A	0.9700	N1—H1B	0.86 (2)
C4—H4B	0.9700	N2—H2A	0.86 (2)
C5—O2	1.244 (3)	N2—H2B	0.86 (2)
C5—O1	1.262 (3)	O3—H3C	0.87 (3)
C5—C6	1.527 (3)	O6—H6C	0.87 (3)
C6—C7	1.537 (3)	O8—H8C	0.87 (8)
C6—H6A	0.9700	O8—H8D	0.87 (8)
			
N1—C1—C2	110.99 (19)	O3—C7—C6	111.33 (17)
N1—C1—H1C	109.4	O3—C7—C10	108.20 (17)
C2—C1—H1C	109.4	C6—C7—C10	108.45 (16)
N1—C1—H1D	109.4	O3—C7—C8	110.29 (17)
C2—C1—H1D	109.4	C6—C7—C8	109.76 (16)
H1C—C1—H1D	108.0	C10—C7—C8	108.75 (16)
N2—C2—C1	110.75 (19)	C9—C8—C7	111.19 (18)
N2—C2—H2C	109.5	C9—C8—H8A	109.4
C1—C2—H2C	109.5	C7—C8—H8A	109.4
N2—C2—H2D	109.5	C9—C8—H8B	109.4
C1—C2—H2D	109.5	C7—C8—H8B	109.4
H2C—C2—H2D	108.1	H8A—C8—H8B	108.0
N2—C3—C4	109.71 (19)	O7—C9—O6	123.3 (2)
N2—C3—H3A	109.7	O7—C9—C8	124.2 (2)
C4—C3—H3A	109.7	O6—C9—C8	112.56 (19)
N2—C3—H3B	109.7	O4—C10—O5	123.8 (2)
C4—C3—H3B	109.7	O4—C10—C7	120.53 (19)
H3A—C3—H3B	108.2	O5—C10—C7	115.69 (18)
N1—C4—C3	110.68 (19)	C4—N1—C1	111.87 (18)
N1—C4—H4A	109.5	C4—N1—H1A	109 (2)
C3—C4—H4A	109.5	C1—N1—H1A	114 (2)
N1—C4—H4B	109.5	C4—N1—H1B	105 (2)
C3—C4—H4B	109.5	C1—N1—H1B	115 (2)
H4A—C4—H4B	108.1	H1A—N1—H1B	102 (4)
O2—C5—O1	123.7 (2)	C3—N2—C2	111.07 (18)
O2—C5—C6	119.9 (2)	C3—N2—H2A	103 (2)
O1—C5—C6	116.4 (2)	C2—N2—H2A	116 (3)
C5—C6—C7	116.23 (18)	C3—N2—H2B	107 (3)
C5—C6—H6A	108.2	C2—N2—H2B	107 (3)
C7—C6—H6A	108.2	H2A—N2—H2B	113 (4)
C5—C6—H6B	108.2	C7—O3—H3C	105 (3)
C7—C6—H6B	108.2	C9—O6—H6C	111 (4)
H6A—C6—H6B	107.4	H8C—O8—H8D	103 (5)
			
N1—C1—C2—N2	−55.1 (3)	C7—C8—C9—O6	−129.7 (2)
N2—C3—C4—N1	57.1 (3)	O3—C7—C10—O4	−4.9 (3)
O2—C5—C6—C7	−20.1 (3)	C6—C7—C10—O4	−125.8 (2)
O1—C5—C6—C7	162.6 (2)	C8—C7—C10—O4	114.9 (2)
C5—C6—C7—O3	51.2 (3)	O3—C7—C10—O5	174.87 (17)
C5—C6—C7—C10	170.11 (18)	C6—C7—C10—O5	54.0 (2)
C5—C6—C7—C8	−71.2 (2)	C8—C7—C10—O5	−65.3 (2)
O3—C7—C8—C9	46.2 (2)	C3—C4—N1—C1	−56.1 (2)
C6—C7—C8—C9	169.19 (18)	C2—C1—N1—C4	55.0 (2)
C10—C7—C8—C9	−72.3 (2)	C4—C3—N2—C2	−58.1 (2)
C7—C8—C9—O7	50.3 (3)	C1—C2—N2—C3	57.4 (3)

### Hydrogen-bond geometry (Å, °)

**Table:**

*D*—H···*A*	*D*—H	H···*A*	*D*···*A*	*D*—H···*A*
N1—H1A···O3^i^	0.85 (2)	2.48 (3)	3.068 (3)	126 (3)
N1—H1A···O4^i^	0.85 (2)	1.99 (3)	2.764 (3)	150 (3)
N1—H1B···O1	0.86 (2)	1.95 (2)	2.806 (3)	174 (4)
N2—H2A···O5^ii^	0.86 (2)	1.86 (2)	2.706 (2)	167 (4)
N2—H2B···O1^iii^	0.86 (2)	1.99 (2)	2.804 (3)	159 (4)
O3—H3C···O2	0.87 (3)	1.92 (4)	2.685 (3)	147 (4)
O6—H6C···O5^iv^	0.87 (3)	1.82 (3)	2.671 (3)	167 (5)
O8—H8C···O2	0.87 (8)	2.00 (4)	2.798 (4)	151 (8)
O8—H8D···O1^iv^	0.87 (8)	2.15 (4)	3.003 (5)	165 (10)
C1—H1C···O7^ii^	0.97	2.47	3.391 (3)	159
C3—H3A···O7^v^	0.97	2.58	3.394 (3)	142
C3—H3B···O8^vi^	0.97	2.41	3.344 (7)	161
C4—H4B···O5^ii^	0.97	2.58	3.274 (3)	128
C6—H6A···O6^vi^	0.97	2.57	3.338 (3)	136

Symmetry codes: (i) *x*+1/2, −*y*+1, *z*−1/2; (ii) *x*, *y*, *z*−1; (iii) *x*−1/2, −*y*, *z*−1/2; (iv) *x*, *y*+1, *z*; (v) *x*, *y*−1, *z*−1; (vi) *x*, *y*−1, *z*.
